# A staged approach to resecting a large rectal polyp using endoscopic mucosal resection and trans-anal endoscopic microsurgery: a case report

**DOI:** 10.1093/jscr/rjaf068

**Published:** 2025-02-19

**Authors:** Bhavna A Guduguntla, Jared Yee, Paul E Wise, Ahmad Najdat Bazarbashi

**Affiliations:** Department of Medicine, Barnes-Jewish Hospital/Washington University in St. Louis, 660 S. Euclid Ave., MSC 8066-22-6602, St. Louis, MO 63110, United States; Division of General Surgery, Section of Colon and Rectal Surgery, Barnes-Jewish Hospital/Washington University in St. Louis, 4590 Children’s Place, Suite 9600, St. Louis, Missouri 63110, United States; Division of General Surgery, Section of Colon and Rectal Surgery, Barnes-Jewish Hospital/Washington University in St. Louis, 4590 Children’s Place, Suite 9600, St. Louis, Missouri 63110, United States; Department of Medicine, Division of Gastroenterology and Hepatology, Barnes-Jewish Hospital/Washington University in St. Louis, 660 S. Euclid Avenue, MSC 8124-0086-09, St. Louis, MO 63110, United States

**Keywords:** complex, polypectomy, combined, surgery, endoscopy

## Abstract

Large rectal adenomatous polyps are not uncommon. Proctectomy sparing interventions are favored when feasible. We present a case of a 62-year-old woman, who presented with diarrhea for several years. Colonoscopy revealed a very large 60 mm rectal polyp, biopsied as tubulovillous adenoma. This was successfully resected using a staged approach with endoscopic mucosal resection and trans-anal endoscopic microsurgery. Endoscopic mucosal resection removed 70% of the lesion with central scarred not amenable to resection but amenable to transanal excision. Pathology demonstrated tubulovillous adenoma with negative margins. Flexible sigmoidoscopy at 6-month follow-up revealed well healed scar without recurrence or residual disease. This demonstrates a staged resection for a large rectal polyp which is minimally invasive and organ preserving.

## Introduction

Large, non-malignant, or early malignant rectal polyps can present significant management challenges, particularly when endoscopic interventions are unsuccessful or limited, as radical resection is usually recommended in the form of lower anterior resection or abdominal perineal resection (APR). However, radical surgery for non-malignant or early neoplastic polyps carries inherent risks, including postoperative complications, such as anastomotic leakage or stenosis, fecal incontinence, and can impact quality of life. Management of rectal polyps has thus shifted to less invasive endoscopic and trans-anal methods, when feasible [1].

The management of rectal polyps through endoscopic and trans-anal techniques has varying success rates and adverse event profiles. Endoscopic methods of polyp resection include endoscopic mucosal resection (EMR) and endoscopic submucosal dissection (ESD). ESD has shown higher en bloc resection rates of 89% compared to 47% for EMR, and complete resection rates of 82% versus 56% for EMR, significantly reducing recurrence to 2% compared to 10% for EMR. ESD, however, has a higher risk of complications, including perforation, which can be seen in up to 5% for ESD cases compared to 0.1% for EMR [2]. Noninvasive rectal sparing surgical treatment alternatives to EMR and ESD typically involve trans-anal endoscopic microsurgery (TEMS). TEMS has become an effective alternative for excising adenomas and early rectal carcinomas that are unsuitable for EMR or ESD, providing notably low adenoma recurrence rates, ranging from 2% to 16%, and superior resection margins, with a lower rate of positive margins [3].

While TEMS is a suitable non-invasive surgical option for the resection of distal rectal polyps, it has a limited role in large hemi-circumferential polyps and in polyps greater than 10–15 cm from the anal verge [4]. When endoscopic resection or TEMS fail, radical surgery is often pursued [5]. However, there is limited data on staged endoscopic and trans-anal resection for the management of large rectal polyps. This approach is non-invasive, organ sparing, and safe. Here, we present a case of a middle-aged woman with a 60 mm rectal polyp, which was successfully resected using a staged EMR followed by a TEMS approach.

## Case description

**Figure 1:**
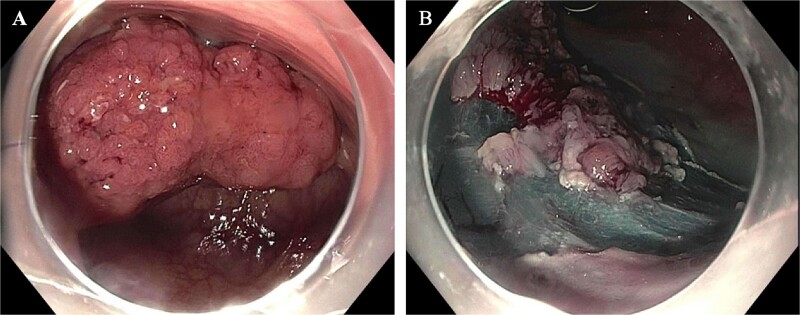
(A) Half circumferential, partially obstructing rectal mass measuring 60 mm in diameter (pictured on the left). (B) Post EMR partially resected polyp with residual central 25 mm polyp.

**Figure 2:**
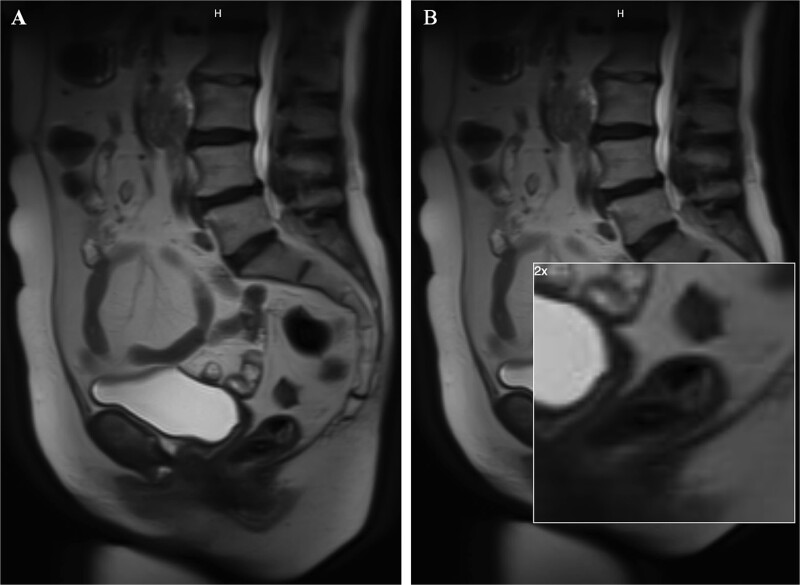
Images from the MRI abdomen pelvis with contrast. This depicts a small polypoid enhancing 2.2 cm mass within the posterior wall of the left mid rectum without invasion of the muscularis propria. The image on the right is a zoomed in image of the remaining polyp.

**Figure 3:**
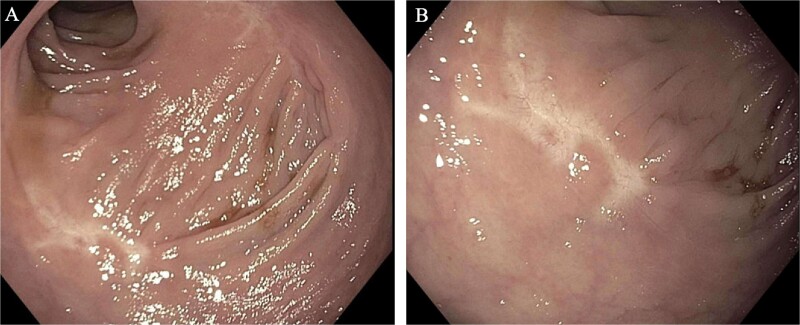
Images from the 6-month follow-up flexible sigmoidoscopy. A large, well-healed scar from the EMR/TEMS resection without residual or recurrent polyp.

A 62-year-old woman with a past medical history of well-controlled type two diabetes, hypothyroidism, and chronic obstructive pulmonary disorder presented with several years of watery diarrhea. She denied abdominal pain, nausea, vomiting, hematochezia, melena, or weight loss. Her physical exam was unremarkable. Laboratory workup was within normal limits. She had not had a screening colonoscopy in the past. She underwent a colonoscopy, which revealed a 60 mm partially obstructing rectal polyp (Fig. 1A). Pathology from initial biopsies confirmed tubulovillous adenoma without dysplasia. The decision was made to proceed with flexible sigmoidoscopy and endoscopic resection. The lesion involved the distal rectal fold and was quite large, making ESD difficult. A decision was therefore made to proceed with EMR. This was successful in removing ~70% of the lesion; however, the central area appeared scarred, and there was concern for deeper submucosal involvement preventing complete resection. This could not be removed with full-thickness resection, cold snare piecemeal resection, or hot snare polypectomy despite further attempts at lifting, ~25 mm of the polyp (the central portion) remained (Fig. 1B); therefore, the patient was referred to colorectal surgery for further evaluation and to attempt completion TEMS. Repeat biopsies at the central residual portions of the polyp revealed tubulovillous adenoma with focal high-grade dysplasia without invasive carcinoma. An MRI of the abdomen and pelvis with contrast was performed several weeks later to evaluate for metastatic disease or deep rectal invasion. Imaging demonstrated a small polypoid enhancing mass within the posterior wall of the left mid-rectum that measured 2.2 × 1.2 cm, without invasion of the muscularis propria (Fig. 2A and B). There were no findings concerning for malignancy or metastatic disease. Decision was made to proceed with TEMS to complete the resection, in hopes of an organ-preserving intervention. She ultimately underwent trans-anal excision of the remaining 25 mm polyp several months later with colorectal surgery. This procedure was uncomplicated. Final pathology showed a 20 mm tubulovillous adenoma, which was completely excised with negative margins and without evidence of residual high-grade dysplasia. At follow-up of 4 months later, the patient reported resolution of her diarrhea. At the 6-month follow-up, flexible sigmoidoscopy revealed a large scar in the rectum without recurrent or residual polypoid tissue (Fig. 3A and B). Biopsies obtained from the scar site confirmed no recurrence.

## Discussion

Colorectal polyp resection is vital in reducing colorectal cancer incidence [6, 7]. Endoscopic resection is typically considered first-line management for small polyps (<5–20 mm) to large polyps (20–40 mm), as it is associated with a lower risk of morbidity and mortality compared to surgical alternatives [8]. Typical endoscopic resection methods include cold snare polypectomy, hot snare polypectomy, EMR, and ESD. EMR is primarily used for rectal polyps >20 mm and can be used for en-bloc or piecemeal resection [9]. ESD is favored when there is superficial submucosal involvement because it preserves the deeper mucosal layers, and if en-bloc resection is favored and cannot be attained with EMR. The risk and method of polypectomy are determined by the lesion size and location, along with its morphological and vascular characteristics and endoscopic expertise [9]. With very large polyps, surgical intervention is typically recommended for definitive excision when endoscopic measures are unsuccessful (higher risk of perforation, incomplete resection, and risk lesion recurrence) [9, 10]. Surgical options for resection include APR, AR, and the less-invasive TEMS. For non-malignant large rectal polyps, the goal is an organ-preserving intervention for improved quality of life, with TEMS preferred over proctectomy if the lesion is accessible and amenable [11]. Limitations to TEMS include very large polyps (greater than half circumference of the rectum) and lesions that are >10–15 cm from the anal verge.

Current literature supports various combination techniques to optimize success with endoscopic resection of high-risk lesions. A meta-analysis evaluated a method using combined EMR and endoscopic full-thickness resection (EFTR) to target colon polyps larger than 20 mm. In 88% of cases there was margin-free disease, and they reported a 2% adverse event rate and 6% recurrence rate at follow-up. However, within the eight studies included in the analysis, the mean lesion size was 35 mm, making it difficult to extrapolate its efficacy to polyps 40 mm or greater [12].

While single-session hybrid laparoscopic/endoscopic techniques are available for polyps in the colon [13], there is little data to support combined advanced endoscopy methods with minimally invasive surgical excision, such as TEMS in the rectum, as employed in our case, particularly as staged fashion. One case report describes a successful single procedure rectal polypectomy with combined ESD using a flexible endoscope and laparoscopic forceps via a trans-anal rectoscopic minimally invasive surgery platform (ARAMIS) [10]. The polyp was 50 mm in length and obstructed 2/3 of the rectum’s lumen. The procedure was uncomplicated, and there was no recurrence at the 50-day follow-up [14].

It is important to note that offering a TEMs at the initial consultation would have been challenging given the lesion’s large size, limiting the ability to successfully remove it and provide tissue apposition for defect closure. EFTR of residual 20 mm lesion was studied, but the lesion was at the upper limit of acceptable size for EFTR and was in a difficult location to allow for it. Therefore, completion TEMS was thought to be the next best step to preserve the rectum and avoid proctectomy.

In conclusion, we present a unique case that showcases a staged model to excise a very large rectal polyp of 60 mm not amenable to sole endoscopic resection, which may otherwise necessitate radical surgery or proctectomy. This approach may prevent the need for radical surgery for similar rectal polyps while preserving the rectum. Further research is needed to evaluate the efficacy of this staged approach on a larger scale, as well as recurrence rates after resection.
